# Summary of Foreign Medical Journals

**Published:** 1865-08

**Authors:** Frank King


### Summary of Foreign Medical Journals.

BY FRANK KING.

Dr. Pagenstecher of Wiesbaden, on the use of the Yellow Amorphous Oxide of Mercury in Conjunctivitis and Corneitis Phlyctenulosa. (*Ophthalmic Review*, July number, 1865.) The author shows that red precipitate—the red oxide of mercury—which is recommended in a large proportion of corneal diseases, is from its crystalline form, inadequate for this purpose, and speaks highly of the almost specific effects of the *yellow amorphous oxide of mercury* in his practice.

"Red precipitate, the red oxide of mercury, has hitherto played an important part in the treatment of the superficial diseases of the eye, in the form of an ointment. When the object of the surgeon was to produce a stimulating effect, in diseases of the conjunctiva and cornea, we find several compound ointments of this kind recommended. The red precipitate is from its crystalline form not sufficiently divided, and does not act uniformly on the diseased membranes, very often, too, gets retained in the folds of the membrane, and there sets up a caustic action. Acting under the advice of Dr. Hoffmann, I substituted the yellow amorphous oxide of mercury. This preparation possesses the advantage of being in the finest possible division, and being altogether destitute of any crystalline form. It differs not only in form, but also in its chemical characters from those which have been hitherto used. Care must be taken in the precipitation to obtain a pure oxide. The precipitation is effected by adding a solution of the chloride of mercury to a solution of potash, in such a way that there is an excess of the latter; after the precipitate has deposited itself, the supernatant fluid is at once poured off, the precipitate thoroughly washed, and dried by a gentle heat, with exclusion of day-light. Thus prepared, the yellow precipitate has a light yellow color, and is an exceedingly fine powder.

The most perfect vehicle for an eye ointment, must be very soft, without however, being too fluid, lest the heavy oxide sink to the bottom; but when in contact with a moderate heat of the body, it must completely melt, aud be quickly and uniformly diffused over the eye. Numerous experiments with hog's lard, butter, glycerine, glycerine ointment and mixed fats, have led me to give the preference to the last, and I recommend either the mixture of spermaceti, wax, or almond oil.

The principal conditions under which the ointment is recommended are conjunctivitis and corneitis phlyctenulosa, which have been called by different authors by the most different names, in mild cases of scleritis, corneitis, vasculosa, corneitis fasciculosa, corneitis ulcerosa or corneitis profunda; the ointment is also an excellent means of clearing the cornea in all those exudations which persist after inflammation. The good effect of the ointment is most displayed in chronic cases, after the originally increased irritation of the cornea has somewhat abated, and the vascularity assumes what is known as passive congestion.

The contra-indications of the ointment are easily known, corneitis purulenta, blenorrhoica, or corneitis vasculosa, originating in granular lids and trachomatous pannus, generally get worse under its use. In syphilitic corneitis parenchymatosa it has no effect; but in the consequent obscurations of the cornea the ointment may be used after all acute symptoms have vanished, to clear the cornea. If any iritis coexists, the ointment must be avoided, as well as in all deep infiltrations and ulcerations of the cornea. Its immediate effect is irritant, and the increased flow of tears and feeling of pain prove that there is no necessity for the application to remain long on the diseased parts to produce its effects. When the ointment is applied for the first time, the irritation may persist for several hours, till gradually it sinks to its previous degrees ; on the second or third application always observing an interval of four-and-twenty hours between each, the immediate irritant effect is always much less. The eye gradually so accustoms itself to it that generally after being used for a week the re-action only lasts for about half an hour. The raw exudation-surfaces lose their rough appearance, their yellowish color gets more grayish-white, and at last they become quite smooth and clear. As regards photophobia, we possess in the yellow oxide of mercury an excellent sedative, due to the previous irritation. It agrees with the more delicate structure of the eye, and only excites that slight amount of irritation which is necessary to exert an alterative action on the diseased tissue, and perhaps exposed, nerves.

The ointment is to be applied but once a day, with a small brush, dipped in it, and applied between the eyelids; if it has the right consistence, it gets by the movements of the lids, diffused over the whole conjunctiva and cornea, and then by the same agency extended from the eye, it should then be wiped off, lest it, by remaining too long in contact, create an undue irritation. If the movements of the lids are insufficient to completely remove it from the conjunctiva, we may effect this by rubbing them together, and raising both the upper and lower lids from the globe. Cold applications after its use generally very quickly subdue the first somewhat violent signs of irritation. If the eye still exhibits any irritation after one or two hours, it disappears after a walk in the open air."

John C. Agnis, M. B., F. R. C. S., Assistant Surgeon Royal Horse Guards, on the treatment of Hydrocele by pressure after injection. (*London Lancet*, August number, 1865.)

After operations for hydrocele, there always remains a certain amount of deformity of the testicle and neighboring tissues. The author of the following treatment has found in his practice that pressure applied in the way usual for orchitis, restores the testicle to a consistency nearly normal. His plan is this, "having tapped the hydrocele, and injected a solution of iodine, he waits till the tenderness has subsided enough for the patient to bear without pain a degree of pressure equal to that produced by strapping. Then he applies the strappings in the way generally done for orchitis; some times the swelling diminishes so fast that the strappings have to be re-applied every second day. He thinks the earlier they are applied the better, provided the inflammation has subsided enough for it to be done without pain."

Samuel Wilks, M. D., Assistant Physician to Guy's Hospital, on Epidemic Cerebro-Spinal Meningitis. (*Lancet*, July number, 1865.)

The author of this article, remarks that the three maladies prevailing in Russia, may be found hereafter to have the same origin, and that one of them, "meningitis spinalis," has prevailed in various parts of Europe and America for many years, in which the main symptoms as well as the deaths were due to an inflammation of the brain and spinal cord. "The petechiæ would denote a blood-disease, and therefore the cerebro-spinal meningitis may be only one of the usual concomitants; since recovery often took place, and an inflammation of the nervous centres is an almost necessarily fatal affection, it can scarcely be regarded as an essential part of the disease. From amongst the author's reports of post-mortem examinations, he records three cases of this disease as occurring suddenly in persons previously comparatively healthy. According to the modern doctrines of pathology, a healthy person cannot be seized with a simple idiopathic inflammation of this kind, it is probable that in these cases there were some accompanying blood-disease, of which the cerebro-spinal meningitis was the most marked outward evidence."

Dr. W. T. Gairdner, Professor of Medicine in University of Glasgow, on Typhus Fever. (*Braithwaite's Retrospect*, July, 1865.)

"In a large proportion of cases, typhus fever left to pursue its natural course, and treated with milk diet and without drugs or stimulants, will have its natural crisis before the twelfth day. No other food can be depended on but milk or buttermilk; to give wine, whisky, and beef tea, while withholding milk is to diminish the chance of the patient's recovery. Large doses of alcoholic stimulants are positively injurious, as is also the deprivation of fresh air. Our guide to the progress of a case of fever is, to watch carefully and constantly the rate of the pulse, for it gradually rises in frequency up to the acme of the fever, and then as gradually declines, so that the crisis can be accurately ascertained. This is of immense importance as regards the prognosis, for the other symptoms may continue as formidable as ever, but if the pulse is gradually declining in frequency, the case will do well."

Frederic H. Morris, M. D., case of Recurrent Fibroid Tumor. (*Lancet*, May number, 1865.)

A brick-maker, aged 42, consulted Dr. Morris about a tumor in the neck. On examination a tumor was found in the parotid region, about the size of a walnut. As his general health was good, removal was recommended. In about ten months he again presented himself; it was now about the size of a lemon, adherent to the fascia of the neck, but the skin was quite movable. He was anxious to have something done, and the operation was performed. No vessels were wounded; the wound healed by first intention, and the patient experienced considerable relief. In a short time another tumor appeared, which rapidly increased in size, and he sank exhausted with pain, etc., about five months after the operation.

On examining the tumor after removal, it presented externally a firm fibrous appearance, the interior was soft and pulpy. In structure the hard parts were essentially fibrous; many of the fibres appeared made up of cells, closely applied together, and in the soft parts isolated cells and nuclei abounded.

The recurring fibroid tumor forms a kind of connecting link between the innocent and malignant formations, and it is an important circumstance that the later-produced tumors approximate much more in appearance and behavior to the malignant character than the original.

